# Codevelopment of a complex intervention to reduce inequalities in paediatric diabetes secondary care outcomes for children with type 1 diabetes from underserved groups

**DOI:** 10.1136/bmjopen-2024-089372

**Published:** 2025-05-06

**Authors:** Aidan Searle, Jessica R Wheeler, Ian Litchfield, Julian P H Shield, Timothy Barrett, Sheila Greenfield, Kerry Leeson-Beevers, Sabi Redwood, on behalf of the Diversity in Diabetes Consortium Members, C Austin

**Affiliations:** 1 NIHR Biomedical Research Centre - Diet and Physical Activity Theme, University of Bristol, Bristol, UK; 2 Bristol Medical School, Population Health Sciences, University of Bristol, Bristol, UK; 3 Institute of Applied Health Research, University of Birmingham, Birmingham, UK; 4 University of Bristol, Bristol, UK; 5 Institute of Child Health, University of Birmingham, Birmingham, UK; 6 Alström Syndrome, London, UK

**Keywords:** Health Services, Person-Centered Care, Self-Management

## Abstract

**Abstract:**

**Objectives:**

To codevelop (with children and young people with diabetes (CYPD)) an intervention to improve diabetes control and future health outcomes of CYPD from ‘underserved’ groups, to reduce treatment outcome inequalities between different socioeconomic and ethnic groups. To follow Medical Research Council guidance for complex interventions and the COM-B (Capability, Opportunity, Motivation, Behaviour) model for behaviour change intervention development.

**Design:**

In phase 1 (previously reported), we established the evidence base, conducted literature reviews and analysed data from semistructured interviews with CYPD and their carers. In phase 2 (this report), we applied the COM-B framework to identify intervention components; in phase 3 (this report), we evaluated these components, including focus groups with CYPD, their carers’ and healthcare practitioner (HCP) surveys, using the Acceptability, Practicability, Effectiveness, Affordability, Spill-Over Effects, Equity criteria.

**Setting:**

Secondary care; children, young people and their carers’ were approached from two large paediatric diabetes services in England, both with socioeconomically and ethnically diverse underserved populations; paediatric diabetes HCPs were surveyed across four English regions.

**Participants:**

N=69 underserved CYPD (aged 5–19 years) and/or family members took part in interviews; N=48 paediatric diabetes HCP survey respondents (survey 1); N=34 paediatric diabetes HCP survey respondents (survey 2); N=3 young people’s advisory group participants; N=17 underserved CYPD/carers focus group participants; N=9 wider stakeholder participants.

**Results:**

The codevelopment process and integration of COM-B established four elements for an intervention package: (1) an enhanced peer support/mentoring programme; (2) provision of a health and well-being coach to CYPD/families; (3) family/community support to address social and community issues and (4) training for HCPs, including cultural competence, poverty proofing and to emphasise the need for increased sensitivity and better supported communication in work with CYPD from underserved groups.

**Conclusions:**

The Diversity in Diabetes codevelopment work informed an intervention to improve diabetes care in underserved groups, reflecting sociocultural contexts and plausible support options at the individual, community and clinical levels. The ‘Diversity in Diabetes’ programme will next test feasibility and further refine the intervention package in two more paediatric diabetes centres in England.

**STRENGTHS AND LIMITATIONS OF THIS STUDY**

- Engagement, inclusion and oversight through a dedicated young people’s advisory group that included children and young people with type 1 diabetes (CYPD) from relevant socioeconomically and ethnically diverse ‘underserved’ backgrounds.
- Included data from in-depth semistructured interviews and focus groups with underserved CYPD and their carers.
- Input from a wide range of stakeholders, including CYPD and their families, and multidisciplinary paediatric diabetes health and social care practitioners.
- The ‘affordability’ component of Acceptability, Practicability, Effectiveness, Affordability, Spill-Over Effects, Equity may impact on the ability of discrete health service teams to adequately attend to communication support needs and entrenched structural socioeconomic disparities.

## Introduction

 Health inequalities are defined by the National Health Service (NHS) as ‘unfair and avoidable differences in health across the population and between different groups within society’.^1 2^ Children and young people with diabetes (CYPD) from black African, South Asian and Caribbean-heritage and/or families living in socioeconomic disadvantage, hereafter ‘underserved groups’, tend to have poorer health outcomes related to diabetes control, compared with CYPD from less socioeconomically disadvantaged and/or white UK populations.^3^ These underserved groups show variation in engagement with structured support and attainment of diabetes-related health targets that are associated with increased health inequalities in outcomes.^4^

Few interventions have targeted self-management for CYPD from underserved groups, with many trials recruiting homogeneous samples from CYPD populations with lower risk of poor health outcomes, and without attending to deprivation or culturally mediated concerns—potentially leading to intervention developments that risk widening inequalities.^5^ Additionally, past approaches to improving diabetes control may have focused attention and attribution of blame on CYPD and their carers’, rather than on the nature of the therapeutic relationship.^6^ There is a recognised need for patient-centred approaches, which focus on therapeutic alliance; appreciation of each CYPD as a whole person; the importance of individual preferences; social and cultural contexts and empowerment.^7^

### Diversity in diabetes study

The ‘Diversity in Diabetes’ programme (NIHR202358) set out to codesign and trial a targeted intervention, sensitive to the needs and preferences of CYPD from underserved groups, in order to improve glycaemic control (routinely assessed for all CYPD in UK paediatric diabetes clinics, through the measurement of glycated haemoglobin (HbA1c).^4^ The Medical Research Council (MRC) framework for developing complex interventions stipulates four phases: (1) identification of the intervention, (2) feasibility, (3) evaluation and (4) implementation.^8^ The present paper offers an overview of phases 1–3 of this process and the outcomes. Phase 1 summarises work that established the evidence base, including preliminary review work and novel empirical work (work that is published, or in process of publication in full detail, elsewhere).^9^,^11^ Phase 2 reports the Capability, Opportunity, Motivation, Behaviour (COM-B) intervention codesign process. Phase 3 reports the stakeholder evaluation. Phases 2 and 3 are presented for the first time in this report.

#### Phase 1: establishing the evidence base—systematic and qualitative evidence synthesis findings

Phase 1 comprised a series of systematic reviews to explore best practice in designing and embedding culturally and socially sensitive, diabetes self-management support programmes.^9 10^ This work found that self-management support programmes needed to retain an appropriate level of context-specific flexibility, achieved throughout the codesign process. Additional review work (a qualitative evidence synthesis) focused on studies of the views and experiences of CYPD from underserved groups, to better understand how these might be integrated in the codevelopment of a diabetes management support programme.^11^ The qualitative evidence synthesis established three analytical themes (alienation, empowerment and integration). A need was identified to focus on diabetes support interventions that would be able to foster greater engagement and better integration of diabetes management through a broader, more holistic, focus on a person and their life, within and beyond the clinical encounter.

#### Novel empirical codevelopment work

Phase 1 also involved novel empirical work; primarily an in-depth qualitative interview study undertaken through the lens of the burden of treatment theory (BoTT).^12^ The BoTT is a model of healthcare that depicts ‘treatment’, especially in the context of chronic health conditions, as a multicomponent relational process, whose outcomes and successes involve substantial and sustained work, not just by healthcare providers, but especially by patients and carers in the form of treatment adherence, self-management and self-advocacy to ensure ongoing treatment and service provision, local adaptations and support needs. The work required by patients and carers is recognised as an unevenly distributed burden, which can be overwhelming (resulting in poor or worsening health outcomes), dependent on the nature and extent of a person’s ill health; the requirements of treatment and self-management; personal and social resources (eg, physical, emotional, social, environmental and financial living circumstances; cognitive and social skills and capacities; carer resources within their wider network of support). This situates treatment outcomes in the context of the patient’s individual lives and focuses attention on a range of personal and contextual resources, recognised as key mediators, relevant to what is (actually) offered, received and used in relation to standardised healthcare provision. Qualitative interviews were analysed in relation to a series of conceptual models that underpin the BoTT (eg, mobilising capacity; expressing capacity; mobilising for delegated tasks; enacting delegated tasks; and interventions that link capacity and work).^12^ Altogether, phase 1 studies (qualitative evidence synthesis and empirical work) recognised a biomedical ‘diabetes discourse’ present within clinical consultations, characterised by a narrow focus on optimising HbA1c measures and a lesser emphasis on individual socioemotional, contextual, behavioural and holistic factors.

#### Phase 2: the COM-B codevelopment process

The COM-B framework was used to help specify links between underserved CYPD contexts, diabetes care and treatment challenges, and relevant behavioural change, using an iterative, consensus-focused coproduction approach, incorporating contributions from CYPD, carers and other key stakeholders.

COM-B is an evidence-based framework for understanding and changing behaviour that refers to three core factors that are necessary and sufficient for any behaviour (B) to occur^13^:

- Capability (C), which can be physical or psychological (knowledge, skills, memory, attention, decision-making processes and behaviour regulation);.
- Opportunity (O) refers to factors in the physical or social environment (environmental contexts and resources and social influences) which impact on behaviour.
- Motivation (M), which can be reflective (conscious or planned) or automatic (unconscious, emotional responses, impulses, habits) and relate to social and/or professional roles and identities, beliefs about capabilities and consequences, optimism, intentions, goals, reinforcement and emotions.

COM-B posits that these factors interact to shape behaviour, and modification of just one of the factors holds the potential to change behaviour. Given prior evidence, the COM-B framework can be used to help identify the most appropriate target(s) for intervention development.

Overall, the paper provides an overview of the whole multicomponent intervention codevelopment process.

## Design and methods

**Figure 1:**
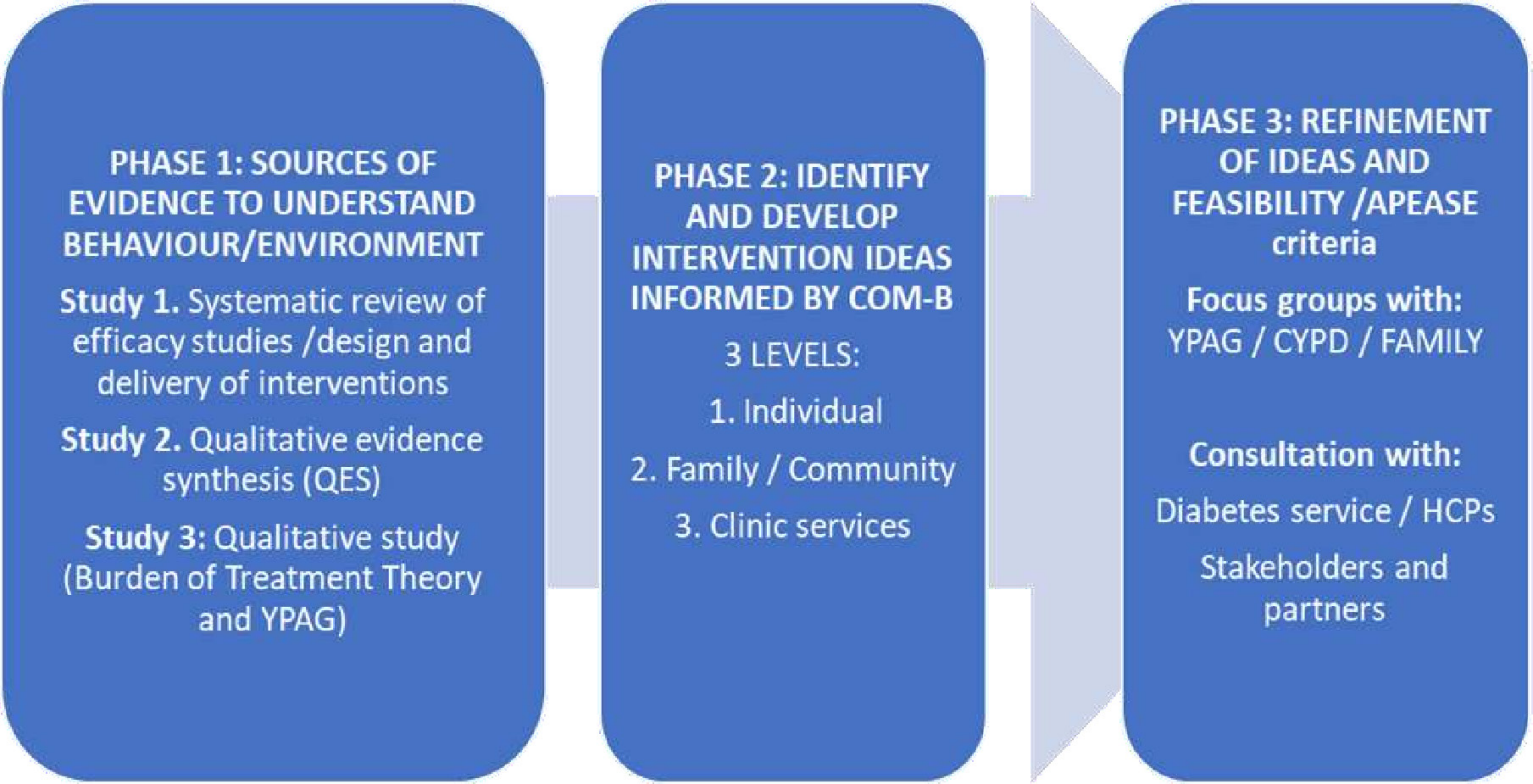
Three phases of intervention design and development. COM-B, Capability, Opportunity, Motivation, Behaviour; CYPD, children and young people with diabetes; HCP, healthcare practitioner; YPAG, young people’s advisory group.

This report provides an overview of three phases of intervention codevelopment work (aligned with the phases set out in the MRC framework for developing complex interventions, depicted in figure 1.

### Patient and public involvement and engagement

Patient and public involvement and engagement (PPIE) was integral to all three phases of the intervention codevelopment process. Our PPIE lead (KL-B) and Diabetes UK partners (CA) were integral to the study design and protocol development. At phase 1 of the development process, a young people’s advisory group (YPAG) was formed (with 12 active members to date) that included young people from diverse/underserved backgrounds, prioritising inclusion of young people with type 1 diabetes and their siblings from underserved communities. The Diversity in Diabetes YPAG informed questions, issues, topics and processes of enquiry throughout. In phases 2 and 3 of the intervention codevelopment process, additional family groups (parents and siblings of underserved CYPD) and wider stakeholder groups were brought into the codevelopment process.

### Phase 1 methods: reviewing the evidence base and novel primary empirical work

1. Review work to explore best practice in the design of culturally sensitive diabetes self-management support programmes included an initial selection of 869 articles, of which 34 were included in two narrative synthesis reviews.^9 10^ Additionally, a qualitative thematic synthesis^14^ was conducted with a focus on the healthcare experiences of CYPD from underserved groups, which located 3849 articles, of which 7 were included in the review.^11^
2. Novel empirical work involved in-depth semistructured interviews with underserved CYPD and family members. This study included CYPD and/or their parents with: (1) UK minority ethnic heritage (including African heritage, South Asian heritage, Black British, Caribbean heritage, mixed heritage—but not white European) and/or CYPD living in the most deprived 20% of neighbourhoods in England (established by index of multiple deprivation score, using postcodes and published datasets^15^ and (2) CYPD (aged 5–19 years) and/or family members of CYPD. CYPD were identified from patient databases, by clinical teams, at two large secondary care paediatric diabetes services in England, both with diverse local populations. Those identified were informed about the study during routine clinical appointments, and with their agreement were contacted by the research team who provided further information about the study. CYPD and/family members who gave their consent (for under 16s assent plus parental consent, for over 16s CYPD consent) were interviewed at home, online, or in an alternative community location (according to their preference) by two experienced qualitative interviewers. Interviews were transcribed verbatim and coded, initially inductively (using NVivo V.14 software to support transcript coding), latterly also deductively, in relation to underpinning components of the BoTT.^12^ Codes were developed analytically, using the methods of thematic analysis,^16^,^18^ with particular consideration given to relationships with components of the BoTT model and to potential areas of focus for intervention development. This work took a critical realist ontological stance,^19^ seeking to identify and interpret patterns in CYPD and carers’ accounts of their experiences, thoughts, feelings and actions, to inform the codevelopment of interventions aimed at behavioural change (enhanced self-management) and biomedical (blood glucose regulation measured through HbA1c) outcomes. Analytic themes were regularly reviewed by and developed with members of the Diversity in Diabetes’ YPAG and wider research team.

### Phase 2 methods: identifying and developing intervention ideas—application of COM-B

The themes identified in phase 1 were mapped onto relevant components of the COM-B framework, to identify and inform appropriate targets for intervention. Intervention targets (COM B ‘intervention functions’ or strategies for behaviour change) mapped on to psychological and physical capabilities, social and environmental opportunities, reflective motivations and automatic motivations.^13^ Codevelopment work (involving facilitated online meetings with the wider research team and the Diversity in Diabetes’ YPAG) focused on phase 1 findings and proposed COM-B intervention targets. Intervention targets were any plausible additional social, emotional and physical support mechanisms or structures, able to bring about change in capability, opportunity or motivation relevant to diabetes management behaviours—in the context of the social and/orfamily life of underserved CYPD, their secondary care clinical consultations and standard multidisciplinary support/treatment.

### Phase 3 methods: evaluation of stakeholder views of intervention

Intervention ideas were refined and evaluated in the context of stakeholder views and APEASE (Acceptability, Practicability, Effectiveness, Affordability, Spill-Over Effects, Equity) criteria.^13^ Phase 3 of the codevelopment process started with consultations (online meetings) with the Diversity in Diabetes YPAG and further underserved CYPD/family (parent/sibling) focus groups. To evaluate preferences and appropriate targets for intervention, findings and intervention proposals were presented at core research team meetings; a wider stakeholder meeting; and within paediatric diabetes healthcare practitioners (HCPs).

#### Consultation with Diversity in Diabetes YPAG and/or underserved CYPD and/or carers

Focus group consultations were conducted online at times that were convenient to participants, with CYPD and/or parents/family/carers who had participated in the primary qualitative data collection (phase 1) and/or YPAG meetings and were keen to contribute to intervention codevelopment work. CYPD/families were presented with and reviewed provisional phase 1 and phase 2 findings and offered additional insights, experiences, ideas for intervention targets and concerns regarding proposed intervention elements targeted at the individual, community and clinic level.

#### Consultation with HCPs: intervention preferences

HCP survey 1: An anonymous online survey of HCPs surveys (using JISC online surveys, with questions and intervention proposals and a variety of tick box and free text answer options) was circulated electronically (by email, including a link to the electronic survey) to HCP and social care practitioners from multidisciplinary paediatric diabetes services via four UK regional children’s diabetes networks. The survey aimed to capture panel members’ previous experience, expertise and views on the proposed intervention strategies generated in phase 2; and to rank the strategies to support intervention codevelopment work. A second anonymous online survey circulated electronically by email to HCPs via the same four regional paediatric diabetes multidisciplinary team networks, focused on eliciting preferences for the three levels of proposed interventions identified in phase 2. This survey of HCP preferences was combined with the responses from CYPD/family focus groups, stakeholder and partner consultation meetings, to gain further clarity about the acceptability and feasibility of the intervention approaches.

#### APEASE criteria

For each proposed intervention element, HCP survey questions were framed with regard to (a) the extent to which it might be relevant/useful for CYPD from underserved groups in their clinic and were guided by criteria of the APEASE framework. The focus at this stage was on three key APEASE criteria: (1) Acceptability—how acceptable the intervention approach is to the HCPs and CYPD/families; (2) Practicability—how easily the intervention element would fit into their current service provision and CYPD/families lives; what changes would the clinic need to make to enable implementation; and whether the intervention should be provided by an external agency or as part of the multidisciplinary clinical team and (3) Equity—to what extent would the intervention element address inequalities in service provision. The remaining criteria were set aside for future evaluation as part of formal feasibility study and randomised controlled trial stages of development (effectiveness, affordability, side effects/safety), though affordability remained relevant to consideration of proposed interventions as a facet of acceptability, practicability and equity. Phase 4 (of the MRC complex intervention development process)—implementation—is the subject of ongoing trial-development work.

## Results

### Participants in phase 1 qualitative study

N=69 underserved CYPD and/or family members took part in interviews (see table 1); N=48 paediatric diabetes HCP survey respondents (survey 1); N=34 paediatric diabetes HCP survey respondents (survey 2); N=16 YPAG members; N=17 underserved CYPD/carers focus group participants; N=9 wider stakeholder participants. HCP survey responses were anonymous, but survey 1 gave an option for practitioners to specify their role: 50% stated medical/doctors; 27% nurses, 12.5% psychology and 10.4% dietetics; survey 2 did not include this option but was circulated via the same four paediatric diabetes practitioner regional networks.

**Table 1:** Phase 1 qualitative interview participants

Characteristics of participants and interviews	Interviewees (N=69)Interviews (N=43)
Age of CYPD	
Under 10 years	9
11–15 years	25
16–19 years	9
UK minority heritage of CYPD and family (however, these are summary categories, participants identified in many different ways for example, British, Black British, Pakistani, Somali).	
African heritage	14
South Asian heritage	11
Caribbean heritage	4
South American heritage	1
Mixed	3
CYPD and family who were eligible by postcode deprivation indices, but not minority ethnic status	
White	10
Gender CYPD	
Male	15
Female	28
Non-binary	–
Gender CYPD parent interviewees	
Male	7
Female	19
Non-binary	–
Living in 20% most deprived neighbourhoods in England	
CYPD and families (including white and minority ethnic status)	30
Total interviews*	43
Total participants	69

* Interviews include those with CYPD alone, with parents alone or with CYPD and parents.

CYPD, children and young people with diabetes.

The findings of the phase 1 qualitative interview study, phase 2 COM-B mapping process and phase 3 consultation and refinement process are outlined below:

### Phase 1: qualitative empirical work—summary of analytical themes

Relevant analytical themes (relating to BoTT and COM-B intervention functions) were (1) the individual and their emotional relationship with diabetes; (2) close family as the primary relational network of diabetes management and pressures on family life, including issues of status, racism, low income and poor housing; (3) the local community (including social services, schools), CYPD/family’ and community relationships, communication, self-advocacy and assertiveness skills and experience, related to local responses and adaptations and (4) the clinic as a strong source of support for diabetes management, and the impact of harsh, negative and invalidating clinical feedback.

### Phase 2: COM-B results

The COM-B mapping process identified a range of potential targets for intervention (COM-B intervention functions or strategies for behaviour change) that focused on modelling, education, persuasion, enablement, and training pathways at the individual, family/community and clinical levels (onlinesupplemental files IIII).

1. *Individual level* (focused on work directly with the CYPD/their parent for younger CYPD); creating the opportunity for CYPD to increase their agency and capability to integrate effective diabetes management behaviours (eg, nutrition, carbohydrate counting, blood glucose monitoring and insulin intake to support daily activities and well-being), and to make use of recommended technological advances (continuous blood glucose monitoring and insulin delivery technologies), in the context of their personal lives and everyday experiences. Diabetes management requires reflective motivation to undertake regular necessary blood glucose control activities. Underserved CYPD and parents did not lack motivation: they wanted to achieve good blood glucose regulation and to avoid future physical harms; they reported thinking about diabetes management often (or indeed ‘all of the time’) and being deeply concerned about their/their child’s future health. Barriers to good self-management related strongly to inhibitors of opportunity, agency and capability, and to emotional factors that undermined their motivation. Underserved CYPD identified overwhelming feelings that interrupted necessary diabetes management behaviours, in particular: not wanting to be different from friends or to stand out in class or other social settings; feeling fed up, frustrated and angry with the constant demands of diabetes management; feeling undermined by tiredness, ill health and a sense that sustained self-management efforts were not working; and associated with these feelings sometimes reached a ‘burnt-out’, reactive, rejection of diabetes and its associated management tasks (eg, not checking blood glucose levels and bolusing insulin as regularly as needed, resulting in poor blood glucose regulation and at worst diabetic ketoacidosis (DKA) and hospitalisation).
  In a social contexts where others do not have to undertake regular diabetes management tasks, CYPD need strong agency to create suitable opportunities to take care of their diabetes, and capabilities to overcome overwhelming emotions (to deal with stress and burnout in the context of constant diabetes management efforts), and/or to create new habits or adopt new technologies that might less effortfully perpetuate blood glucose regulation.
  It was proposed that an appropriate intervention at the individual level would also serve to alleviate some of the alienation and isolation that CYPD from underserved groups described in relation to their experiences of type 1 diabetes in the context of other forms of marginalisation.
  Selected COM-B intervention functions (strategies for change) focused on modelling, persuasion, enablement, education. Plausible targets were identified as (1) health and well-being coaching to attend to emotions, stress and burnout and to promote greater agency in the creation of opportunities and habits that include emotional regulation practices; and to support better use of advanced diabetes management technologies and (2) peer support to address alienation and isolation and to support sharing, modelling and increased agency (self-confidence and self-efficacy) around self-care.
2. *Family/community level*: The focus here was on engendering support and adaptations to increase parental/child opportunity and capability to attend to diabetes management in everyday life, and to overcome diabetes management obstacles. Within families, tensions can arise as young people become increasingly independent and responsible for their diabetes management, and parents wrangle with reduced control (eg, over diet, activity, blood glucose monitoring and insulin dosing), experience anxieties about the risks of mis-management (eg, DKA, coma or future health outcomes), and want to maintain good relations, rather than battle with their growing child. In wider social networks, parents of underserved CYPD can experience barriers to eliciting support from peers and extended family, where there is poor understanding of type 1 diabetes and its management, resulting in misplaced judgements and poor recognition of the child, young person or family support needs. In the wider community, recently migrated, first generation parents, with English as an additional language do not always find it easy to communicate and assert their and their children’s support needs (eg, to attend a school that is nearer to home; to communicate by phone with a school or clinic to address diabetes management concerns; to fully understand and ask for what is offered by the clinic or other support services; to access income or disability financial supports; to address housing issues or additional support needs of other family members). Selected COM-B intervention functions (or strategies for change) focused on environmental restructuring, training, enablement. Plausible targets for intervention development included (1) family support, including communication enhancement through quality interpreting services; and ad hoc advocacy/supported self-advocacy, and support with communication and mediation with local agencies (eg, ad hoc advocacy/supported self-advocacy work with schools, the clinic or other local community services) and (2) peer support (including peers with shared first language/heritage) to increase agency and support self-advocacy through modelling and sharing of experiences and effective strategies for engagement with local service providers/support resources.
3. *Clinic level*: Here, the focus was CYPD/families’ experiences of clinical interactions and clinicians’ understanding and responsiveness. Our data suggested that routine appointments can have a narrow clinical focus (eg, HbA1c/blood glucose data from monitoring devices/insulin dosing recommendations) and that where diabetes management has been poor, there were experiences of invalidating/judgemental/harsh communication that lacked sensitivity to life events, hardships, family relationships (eg, CYPD acting as interpreters for parents) and sociocultural awareness. CYPD and their families can lack agency in navigating unfamiliar services, for instance, lacking understanding of the kinds or range of psychological support that CYPD/family can receive; and awareness of and access to the latest diabetes technology (eg, especially where parents are not technologically savvy, or do not own the latest ‘smart’ phones). This can impact the quality and effectiveness of multidisciplinary team support offered and received and the diabetes technologies offered or made available to CYPD (eg, the latest insulin pumps and continuous glucose monitoring devices). CYPD and families are liable to a sense of cultural and socioeconomic distance and alienation from practitioners in multidisciplinary clinical teams, where there are few, if any, practitioners from similar backgrounds, and an inadequate appreciation of the personal and social contexts that impede good diabetes control. Selected COM-B intervention functions (strategies for change) focused on training and education within the clinic, to increase sensitivity to CYPD/families social contexts and experiences; to reduce harsh, judgemental and invalidating responses; to promote understanding and access to broad ranging paediatric diabetes services and technologies; and to increase diversity within paediatric teams, to better reflect diversity within local populations and CYPD/families patient populations.

### Phase 3: stakeholder consultation/APEASE

Phase 3 determined the relative acceptability, practicability and equity of the interventions through applying APEASE criteria. Ideas and responses derived from CYPD/families, HCPs, stakeholder and partner consultations led to further refinement and development of the three levels of intervention (online supplemental file IV).

Focus groups and stakeholder consultation work supported increased working with local HCPs on engagement with young people; emotion coaching; enhanced underserved-community focus local peer group work within clinics, and facilitated peer support and mentoring activities. While recognising HCP training as valuable, HCPs did not always prioritise this intervention component, given already available professional training and local expertise in sensitive work with diverse communities. Most HCPs favoured the provision of additional support outside the clinic at the level of the family/community (ie, family support worker) to deliver a more targeted package of care to CYPD and families. HCPs emphasised the need to ensure parents/carers were educated on the risks and consequences of poor diabetes control but also that they could access support where family/social pressures may be undermining diabetes management at home or in the community. HCPs raised concerns around safeguarding and social service referrals and how this can undermine relations between HCPs and CYPD parents/carers, indicating the need for increased social care representation within teams, sensitive communication and enhanced communication and mediation between HCPs and families. Finally, HCPs emphasised that continuous glucose monitoring can be a source of conflict within families in that CYPDs can feel overmonitored by parents accessing their data. Recognition of pressures in the triadic relationship between HCPS, parents and CYPD highlighted the sensitive developmental issues and related negotiations that underpin paediatric diabetes management, in the context of emergent CYPD independence and parental concerns for risks. In raising these issues, HCPs reaffirmed the need for sensitive and inclusive communication and mediation work in the clinic (to support key circles of family support as CYPD take on increasing responsibility for their self-care). Placing common CYPD-parent tensions in the context of family life (and related circumstances, for example, socioeconomic deprivation, community relationships/advocacy and communication), on top of the wider processes and pressures associated with young people’s social and emotional development and growing independence, reaffirmed the potential role of health and well-being coaching and family community advocacy support. Health and well-being coaching was recognised as a means to support CYPD to take on diabetes management responsibilities in the midst of the varied developmental, personal and contextual factors that are shaping their lives, relationships, emotions and daily experiences; and to support family members to deal with their own anxieties and fears; to surrender control for diabetes management; and to find new ways to support their increasingly independent child, in the context of their own additional life pressures.

The outcome following phase 3 was an intervention package of 4 elements, at three levels (table 2).

**Table 2:** Phase 3 – elements of intervention development

**Individual level**	At the individual level, there were opportunities to intervene with two distinct elements outside the routine paediatric diabetes service.
Element 1	Referring CYPD from underserved groups into a structured peer support programme and training in self-advocacy and empowerment.
Element 2	Provision of health and wellbeingwell-being coach training for CYPD/families to attend in particular to emotional regulation in the context of everyday life pressures and diabetes management; to manage relational tensions; to promote agency and self-efficacy.
**Family/Community level**	A community or family support worker to address gaps in current service provision (or to increase the availability of family/community support for underserved families, where family/community support is already available).
Element 3	
**Clinic level**	An opportunity to intervene with an overarching approach to health service staff training to raise awareness of CYPD/family issues that had been exposed in the co-development process.
Element 4	Raising awareness of lived experiences of adversity; increasing cultural competence and through the implementation of HCP training and enhanced communication support in clinic (eg, in-person high -quality interpreting as standard where English is not the preferred language); increasing diversity in the composition of HCPs through employment strategies; shifting the ethos of the paediatric diabetes multidisciplinary team through a focus on the provision of feedback and consultation that is appreciative of CYPD and family experiences, considerate, sensitive, non-judgemental, emotionally validating; and by working with CYPD and their families on appropriate person-centred strategies that identify individual barriers and find appropriate targeted interventions (eg, social, emotional, technological).

CYPD, children and young people with diabetes; HCP, healthcare practitioner.

#### Element 1

An enhanced peer support programme.^20 21^ Focus groups indicated that CYPD were interested in shaping programmes focusing on specific peer-led diabetes issues (eg, adaptation to new technology, feelings of isolation, family dynamics, school and socioemotional issues) as well as engaging social activities (eg, arts/sports/camps). Parents were also interested in accessing facilitated peer support to meet other parents of CYPD with similar backgrounds. Stakeholder/partner consultations highlighted the opportunity to collaborate with similar support programmes in development for CYPD (eg, Diabetes UK’s Together Type 1 programme).^21^

#### Element 2

The potential for health and well-being coaching sessions was identified, aiming to address CYPD or family (parent/carer) everyday concerns, life pressures and goals, emotion regulation and strategies to manage challenges, in the context of diabetes and diabetes management but with a focus on the whole person and balancing personal and social life and challenges with health concerns. Health and well-being life coaching^22^,^24^ sessions are designed to support participants in gaining access to relevant health information; address everyday life concerns (the bigger picture) and support emotion regulation, assertiveness and self-advocacy in the community and with HCPs, and self-efficacy in relation to health management. Central to this work is a focus on addressing feelings, motivations, beliefs, expectations, planning and habit forming to support general health and well-being, including mental health, with an emphasis on balancing everyday life concerns, future goals and health management.

#### Element 3

The roles of family/community support worker and/or youth worker were identified with the potential for addressing social and community issues, where advocacy and allyship might be key to CYPD and families successfully attaining necessary changes (eg, to housing, financial support and benefits, school, social services or healthcare team support) to better adapt their environment to support diabetes management. The aim is for this worker to act as an advocate and well-being support for both CYPD and their families, bridging the diabetes clinic and community (such as liaison with school). The role should take into account local community demographics to enable a culturally sensitive approach to advocacy and support.

#### Element 4

CYPD, families and HCPs recognised the need for positive and sensitive communication. HCPs recognised the importance of cultural and social competence and welcomed specific input related to sensitivity and communication in work with CYPD from underserved communities to enhance partnership working and treatment concordance. HCP concerns related to clinical pressures on time and resources and the need for additional funded time to allow for more sensitive and solution-focused work. In relation to sensitivity gaps in clinical communication, suggested additional input related to ‘cultural competence’, training on positive and sensitive, emotion-validating feedback and ‘poverty proofing’ training.

## Discussion

We have reported the codevelopment of a complex intervention following MRC guidelines^8^ for complex interventions in which multiple data sources were integrated leading to the development of the ‘Diversity in Diabetes’ intervention that is both holistic and person-centred. This work took into account the personal, contextual and clinical worlds in which diabetes control behaviours are enacted, supported and sustained. The COM-B mapping activity^13^ informed the selection of peer support interventions; health and well-being coaching (with a focus on emotion regulation, self-advocacy, assertiveness and self-efficacy, agency and empowerment); and family and community support to strengthen and build relational networks and community adaptations to better support diabetes management.

A focus on peer support, coaching and family support is consistent with the finding that engagement and retention in diabetes support programmes is best achieved through sharing practical experiences and work that builds understanding, self-confidence and skills needed to self-manage.^11 25^ Findings echoed widely acknowledged benefits of peer support and family and community interventions, including health and well-being coaching, to improve health outcomes for underserved groups.^20 23^ Intervention mechanisms include improved access to networks of support; increased engagement in, and knowledge and awareness of techniques, processes, technologies, treatments and supports for improved emotional well-being and health management; increased confidence and self-advocacy.^25 26^ The codevelopment process also highlighted the desire for services to better reflect their local populations so that there is a greater mirroring within the clinic of the backgrounds of CYPD/families. CYPD and families welcomed especially the possibility of increased contact with peers and practitioners with direct experience of type 1 diabetes as well as with shared social, economic and cultural backgrounds.

A recurring theme related to the narrow ‘biomedical model’ that continues to shape and dominate clinical consultations. For there to be effective engagement in diabetes management, the need was recognised for HCPs to have a broader understanding of the lives and experiences of CYPD and their families. The biomedical ‘diabetes discourse’ documented and observed in the qualitative evidence synthesis and described by CYPD and families in interviews, related to a tendency within the clinic to focus overly narrowly on discussion of biomedical data: HbA1c measures; daily blood glucose levels; carbohydrate counting and insulin dosing.^11^ Intervention codevelopment focused on opportunities to broaden and strengthen engagement, understanding, sensitivity and support with diabetes management in the context of a range of additional pressures on the lives of underserved CYPD and their families.

Effective communication was recognised as key. Where social and cultural backgrounds differ from those of the majority of other patients and practitioners, a lack of shared languages and restricted communication can undermine understanding between HCPs and CYPDs. Compromised communication can also reduce awareness of, and access to, appropriate support, even where relevant support might ostensibly be available within the service. Effective communication—that includes discussion of potentially sensitive (socioeconomically or culturally nuanced) life circumstances—can also be undermined by the stigma attached to relatively disadvantaged life circumstances. If good communication is lacking within routine clinical consultations, and if individual circumstances relevant to diabetes management are not disclosed, or are not met with sufficient understanding, appreciation, empathy and sensitivity; then issues that are relevant to diabetes management (capacity, opportunity, motivation and behaviours) may come to be seen by CYPD and families as beyond the concern of the multidisciplinary team. Reducing opportunities to understand the contexts of management difficulties simultaneously reduces opportunities to provide appropriate support.

Impacts on diabetes management that related to material and social circumstances and life events (such as living in dilapidated and overcrowded housing; working long or unsociable hours for low pay; caring for other family members with serious or chronic health conditions; holding precarious immigration or welfare status; experiences of racism; bereavements) were of greater relevance than culturally specific issues (eg, religious practices; cultural food preferences; beliefs about diabetes). Recruiting a range of UK minority heritage participants (CYPD and parents) who participated in focus groups throughout the codevelopment process was invaluable in determining this.

Strengths of this study related to effective engagement and intervention codevelopment with underserved CYPD and family members throughout the research design, empirical data collection and intervention development process. The study was informed by established frameworks, incorporating the MRC complex intervention development framework,^8^ the COM-B and APEASE criteria.^13^ However, it was not possible to assess the full APEASE criteria; intervention codevelopment work focused on acceptability, practicability and equity; further feasibility and trial work is planned to establish affordability, spill-over effects and equity.

### Implications for paediatric diabetes health services

It remains the case that resources available to multidisciplinary paediatric diabetes teams and local services are limited by financial constraints and service pressures. Multidisciplinary team practitioners recognised the need for, and welcomed the possibility of, greater access for CYPD and their families to interventions such as health and well-being life coaching; socially and culturally attuned peer support; family and community support work. These are all areas of concern and burgeoning work within paediatric diabetes services^25^,^27^; however, availability is not uniform across regions or Trusts. If our work serves to strengthen the evidence base and justification for enhanced provisions, there will be a need to amend budgets to allow more uniform and comprehensive provision, across regions and ultimately to provide more holistic support for all CYPD, wherever there are additional support needs and life pressures.

We cannot, from the perspective of secondary health services, hope to directly address the structural inequalities that underpin many disparities in health outcomes (eg, housing, educational opportunities, income, precarity; racism). However, by better understanding the detailed ways in which wider inequalities impact the management of diabetes among diverse, underserved CYPD and their families, we can strengthen the call for dedicated funding to provide targeted evidence-backed interventions that can, we hope, mitigate and help overcome some of the discrete barriers to better diabetes health outcomes. Through this work, we might also have some potential to bring about wider health and life benefits for diverse and underserved CYPD, by promoting enhanced and more sensitive communication, appreciation and respect; greater access to supportive peer networks; better community advocacy and self-advocacy to help access support and bring about needed adaptations and by supporting greater assertiveness, self-efficacy and agency in health management.

## Supplementary material

10.1136/bmjopen-2024-089372 online supplemental file 1

10.1136/bmjopen-2024-089372 online supplemental file 2

10.1136/bmjopen-2024-089372 online supplemental file 3

10.1136/bmjopen-2024-089372 online supplemental file 4

## Data Availability

Data are available on reasonable request.
