# Supplementary material for: Codevelopment of a complex intervention to reduce inequalities in paediatric diabetes secondary care outcomes for children with type 1 diabetes from underserved groups
Source: BMJ Open. 2025 May 6;15(5):e089372. doi: 10.1136/bmjopen-2024-089372 (PMC12056613; doi:10.1136/bmjopen-2024-089372)
Supplement: online supplemental file 1 [file bmjopen-15-5-s001.docx]

**Supplementary files Table I: Application of COM-B (CAPABILITY) in Phase 2 of ‘Diversity in Diabetes’ intervention development**

| **COM-B / SOURCES OF BEHAVIOUR** | **PHASE  1**  **Evaluation / Behavioural diagnosis**  **Evidence: qualitative evidence synthesis / BoTT interview study** | **FOR BEHAVIOUR CHANGE TO OCCUR CYPD WOULD NEED TO:** | **PHASE 2**  **WHAT NEEDS TO BE DONE TO CHANGE BEHAVIOUR / ENVIRONMENT: COM-B** | **FACILITATION OF POTENTIAL INTERVENTION LEVEL**   1. **Individual,** 2. **family/community and** 3. **clinic/ service provision** |
| --- | --- | --- | --- | --- |
| **CAPABILITY – PSYCHOLOGICAL**  Knowledge, memory, attention, decision-making processes and behavioural regulation (CYPD/CYPD family) | Stress, emotions (feeling different/frustration with diabetes), burnoutimpedes capability to enact diabetes management  CYPD/family may lack understanding of health services, and be compromised by the need to communicate in a second or third language, impacting assertiveness and self-advocacy skills and ability to negotiate successfully within the clinic, the family and their wider social environment, in turn impacting on others’ responsiveness, support and the availability of adaptations that support diabetes control behaviours. | Engage in an emotional and social support structure to manage overwhelming emotions and burnout and strengthen capability to engage in decision processes that impact on ability to enact diabetes control behaviours.    Increase awareness and understanding of services and resources that are available; gain support to enhance communication, increase self-advocacy and assertiveness and negotiation skills to access needed resources. Experience greater equity in clinical consultations and access to wider array of supports and adaptations in the clinic and in the wider community. | **MODELLING:** Providing an example for CYPD to aspire to or imitate (e.g. emotional regulation, assertiveness, self-advocacy; e.g. through peer support, mentoring or coaching work).  **PERSUASION:**Using communication to induce positive feeling to stimulate action; reinforce positive efforts; encourage (e.g. coaching, using explanation, encouragement and peer support to show benefits of different approaches, e.g. feeling better, better health outcomes, feeling more in control, more positive about diabetes, less overwhelmed, etc.  **ENABLEMENT:**Increasing means, coaching in strategies and techniques /attending to emotional strains / reducing barriers (e.g. support to enable effective communication) to increase capability and opportunity | **Individual level:**  Increase agency in self-care behaviours through coaching and/or sharing of strategies/ skills  Identify issues and share experiences to promote acceptance of diabetes through potential peer support structures or coaching initiatives. Help CYPD deconstruct emotional associations that are real or perceived barriers to diabetes control behaviours  **Family/ community level**: Potential to enhance  CYPD /Family cohesion and self-advocacy through family support worker role to increase capability to overcome barriers to enacting diabetes control behaviours (increase potential for appropriate adaptations in wider social environment that can support diabetes self-management).  **Clinic level:** explore solution-focused / emotion validating/ person-cantered approaches in clinical consultations to increase psychological capability to enact diabetes control behaviours |
| **CAPABILITY -PHYSICAL**  Skills, abilities or proficiencies acquired through practice | Poor access,  adoption and use of latest and most appropriate technologies to assist carb counting / BG monitoring / insulin administration.  Inconsistencies in dietary advice (e.g. ‘eat whatever you want, just count carbs accurately and take the right amount of insulin’ vs low GI diet recommendations), cultural sensitivity in nutritional advice and healthy eating advice that is related to confusion and sometimes tensions between parents and children, or between families and clinics; inadequate recognition and understanding of some aspects of diabetes nutritional advice e.g. healthy eating, low GI diet recommendations, and lack of discussion of the need for flexibility and rapid adaptability in relation to the impacts of illness, stress, hormones, changes in activity; growth; weather/holidays; and impacts on diabetes management. | Increase knowledge and adoption of technology to support agency in enacting diabetes control behaviours.  Identify and learn how a range of nutritional choices impact blood glucose regulation and wider health.  Recognise patterns of blood glucose self-regulation in relation to different, individual, nutritional, social/environmental contexts. | **TRAINING:**Imparting skills to foster greater knowledge/adaptation to technology; making sure CYPD and families know what is available and how different technologies can make diabetes management easier resulting in better regulation; examples from peers to address fear of new and unknown technologies. Clinical support with access to available technologies where family income is a barrier.  **ENABLEMENT:**Increasing means (access to latest technology, including for those with low income and unable to afford latest smart phones) / reducing barriers to capability and opportunity (showing how technology works where CYPD/parents are unfamiliar or are not tech savvy; increasing discussion time in clinic on tech options, and opportunities to trial new tech or to talk to peers who have successfully adopted new technologies).  **EDUCATION:**Increasing knowledge or understanding, including around diet, nutrition and various choices available to support blood glucose regulation, health, well-being and recognise the ‘shifting sand’ of day to day biophysiology in response to ill health, stress, hormones, growth, weather, activities etc. and to support increased agency in experimentation. | **Individual level:**Have access to information and resources regarding technology to promote blood glucose regulation. Support and coach skills to address fears and anxieties, and improve capability to use technology effectively. Identify and create opportunities to practice making diet and lifestyle decisions to optimise blood glucose regulation.  **Clinic level / Service provision:** Promote self-care through adaptation to new technology and support CYPD to integrate diabetes control in the broader context of their lives, including appreciation of changes in health, diet, activity, hormones, growth, weather.  Improve communication, increase capability to ask questions to gain greater understanding of individual diabetes management concerns. Setting realistic shared goals to test new strategies and overcome barriers to effective blood glucose regulation in home and social environments.  Address technology access and uptake. Provide regular reviews of nutritional education to avoid reliance on fixed, rather than flexible, approaches. |
