# Supplementary material for: Codevelopment of a complex intervention to reduce inequalities in paediatric diabetes secondary care outcomes for children with type 1 diabetes from underserved groups
Source: BMJ Open. 2025 May 6;15(5):e089372. doi: 10.1136/bmjopen-2024-089372 (PMC12056613; doi:10.1136/bmjopen-2024-089372)
Supplement: online supplemental file 2 [file bmjopen-15-5-s002.docx]

**Supplementary files Table II: Application of COM-B (MOTIVATION) in Phase 2 of ‘Diversity in Diabetes’ intervention development**

| **COM-B / SOURCES OF BEHAVIOUR** | **PHASE  1**  **Evaluation / Behavioural diagnosis**  **Evidence: qualitative evidence synthesis / BoTT interview study** | **FOR BEHAVIOUR CHANGE TO OCCUR CYPD WOULD NEED TO:** | **PHASE 2**  **WHAT NEEDS TO BE DONE TO CHANGE BEHAVIOUR / ENVIRONMENT: COM-B** | **FACILITATION OF POTENTIAL INTERVENTION LEVEL**   1. **Individual,** 2. **family/community and** 3. **clinic/ service provision** |
| --- | --- | --- | --- | --- |
| **MOTIVATION - REFLECTIVE**  Beliefs about capabilities, optimism, beliefs about consequences | CYPD often describe high motivation to engage with diabetes control behaviours, however, there is also a high emotional load involved in the effort to consistently perform diabetes management tasks that socially differentiate them from peers. Poor diabetes management is associated with poor health outcomes that reduce CYPDs capability to enact diabetes management behaviours (this is a vicious cycle). However, CYPD report thinking about diabetes all the time and a sense of overwhelm (diabetes burnout) that sometimes leads to avoidance of diabetes management; motivation is overwhelmed by frustration, annoyance, exhaustion, a sense of failure, and other emotional responses to diabetes. The stress and strain associated with undertaking regular diabetes management can be exacerbated by poor support and understanding from authority figures such as parents/community and HCPs and by harsh, judgemental and emotionally invalidating feedback. | Consider if and how socio-emotional concerns become barriers to diabetes self-care. Validate, understand and address emotional concerns, overwhelm, exhaustion and burnout.  Discuss emotional responses and related personal/contextual concerns as part of diabetes consultations and make these the focus of consultations where necessary to attend to underlying causes of poor diabetes management. | **PERSUASION:**To enhance self-efficacy / agency and diabetes management, to try new strategies to address emotional barriers that inhibit diabetes management (despite motivation to manage diabetes).  **ENABLEMENT:**Assist problem solving to address context specific barriers, especially burnout, emotional strain and stress.  Facilitate activities that promote self-efficacy/ agency; responding to emotions and techniques to regulate emotional responses to diabetes management.  The use of technologies that reduce emotional load and effort involved in effective self-management.  **TRAINING:**Imparting skills and knowledge with sensitivity to social and cultural contexts and pressures of everyday life. | **Individual level:**Offer relevant and evaluated approaches to supporting CYPD in socio-emotional issues as they arise through provision of CYPD peer support structures and coaching approaches that focus on emotions, stresses, and beliefs about capabilities in this context.  **Clinic level / Service** provision: focus on issues that CYPD may be experiencing in their diabetes management (e.g. increased psychology support attending to emotional pressures and personal life circumstances and enhancing self-efficacy/ agency beliefs that hinder acceptance of diabetes as part of self-identity). Address self-identity, stigma and shame and current developmental issues of CYPD  Offer  ‘cultural competence’  training. Paediatric diabetes HCPs provide positive supportive feedback, without adopting judgemental ‘success or failure’ narratives, or loading shame or stigma onto diabetes management experiences of CYPD and family. Setting of shared goals for blood glucose regulation with awareness and sensitivity of individual social and cultural pressures and impacts of low income on CYPD and family, recognising efforts and achievements and attempting to increase availability of additional support/resources where this is needed and wanted. |
| **MOTIVATION – AUTOMATIC**  Reactions driven by unconscious internal processes e.g. habits, drives, desires, impulses, inhibitions | Exogenous social and / or school environment can impede engagement with diabetes management behaviours such as regularity of blood glucose monitoring/insulin dosing or appropriate nutrition/dietary intake.  Recognition of endogenous and automatic shifts (e.g. in energy/tiredness/mood/ motivation, especially when blood glucose is low) may be overlooked. CYPD and family may lack strategies to recognise and attend to emotional overwhelm as a barrier to diabetes management. | Develop strategies which will help in establishing new nutritional and diabetes management habits and patterns, that reduce the need for reflective motivation and are automatic and so less effortful (e.g. improved technologies that can support more regular and finer-tuned blood glucose monitoring and insulin dosing adjustments).  Attempt to bring automatic emotional responses (e.g. related to long term efforts of diabetes management, or to daily shifts in mood associated with blood glucose levels) into more reflective awareness, to enable the use of strategies to support decision-making to regulate emotions or manage blood glucose (e.g. recognition of emotional responses that can restrict ability to manage diabetes effectively and impact of blood glucose on emotional responses). | **ENVIRONMENTAL RESTRUCTURING:**Changing the physical and social context – increasing potential to bring diabetes management under reflective control, and/or increasing the automatization of behaviours that are otherwise effortful.  **TRAINING:**Gaining new skills e.g. in emotion recognition and regulation.  **PERSUASION:**Using communication to generate positive feeling and stimulate diabetes control  **ENABLEMENT:**Assist problem solving to address overcoming context specific barriers – new strategies to recognise and attend to social and emotional contexts.  Facilitate activities that promote self-efficacy/ agency – recognising circumstances that undermine diabetes management and activities that can address these. | **Individual level:**Provide coaching on navigating the social and emotional environment to aid habit formation (e.g. emotional recognition and regulation; appropriate use of new technologies) and/or habit reversal (emotionally reactive diabetes behaviours that compromise diabetes management; habitual use of outmoded diabetes technologies that are not best supporting diabetes management) to increase integration of diabetes in daily life through better understanding, incentives/rewards for change and awareness of emotional and physiological states that can undermine diabetes management.  Offer prompts and tips on how to develop new habits (e.g. association, repetition) for that support automatic or reflective diabetes management behaviours.  Prompt to restructure home and social environment to promote new habits (e.g. to make use of new technologies, to use emotion regulation strategies). |
