# Supplementary material for: Codevelopment of a complex intervention to reduce inequalities in paediatric diabetes secondary care outcomes for children with type 1 diabetes from underserved groups
Source: BMJ Open. 2025 May 6;15(5):e089372. doi: 10.1136/bmjopen-2024-089372 (PMC12056613; doi:10.1136/bmjopen-2024-089372)
Supplement: online supplemental file 3 [file bmjopen-15-5-s003.docx]

**Supplementary files Table III: Application of COM-B (OPPORTUNITY) in Phase 2 of ‘Diversity in Diabetes’ intervention development**

| **COM-B / SOURCES OF BEHAVIOUR** | **PHASE  1**  **Evaluation / Behavioural diagnosis Evidence:**  **Qualitative evidence synthesis / BoTT interview study** | **FOR BEHAVIOUR CHANGE TO OCCUR CYPD WOULD NEED TO:** | **PHASE 2**  **WHAT NEEDS TO BE DONE TO CHANGE BEHAVIOUR / ENVIRONMENT: COM-B** | **FACILITATION OF POTENTIAL INTERVENTION LEVEL**   1. **Individual** 2. **family/community and** 3. **clinic/ service provision** |
| --- | --- | --- | --- | --- |
| **OPPORTUNITY Physical / Environmental (Context and resources)**  To what extent do physical or resource factors facilitate or hinder self-management? | Families with low income and /or living in area of deprivation can impact opportunity to prioritise diabetes management and make informed / healthy choices (e.g. other children, wider family concerns, health issues, low income, housing issues).  Parents’ working hours may restrict attention to CYPD, and may have multiple dependents to care for.  Reliance on restricted diets (e.g. a set volume of pasta, where carbs are easily identified) and limited review of evolving nutritional guidance or changing lifestyle preferences/nutritional needs. | Access financial / disability/ housing benefits and other supports to increase ability to give time/attention/focus to CYPD and diabetes management. | **ENVIRONMENTAL RESTRUCTURING:** Changing the physical and social context (e.g. improved housing, reduced financial pressure).  **TRAINING:**Facilitate activities that promote self-advocacy to access available support/resources..  **ENABLEMENT:**Increasing means / reducing barriers to increase capability and opportunity (eg through disability benefits that reduce the need for additional parental working hours, or through dedicated work with CYPD to increase ability to attend independently to diabetes management. | **Family / community level:** offer guidance on claiming Disability Living Allowance and in preparation for changing benefits regulations at age 16/support with benefits claims/ and housing issues, signposting appropriate family support resources and navigation of social care and council systems and community support organisations.  Revisit strategies for healthy eating, including on a budget and taking into account cultural dietary preferences.  **Clinic level:** focus on use of language, interpretation/ translation issues and  ‘culturally competent’ and empathetic practice  Shared-decision making for realistic / achievable goals in the context of CYPD/ families social and financial circumstances. Strategies for CYPD /older siblings (where appropriate) to take on diabetes management independently of parents if/when needed and to increase the circle of effective support for diabetes management.Regular review of diet and nutrition advice to ensure flexibility and clarify guidance as CYPD grows and as lifestyles change. |
| **OPPORTUNITY – SOCIAL**  Social influences, social pressure, norms, conformity, social comparison power relations, group identity) | Management of diabetes requires CYPD to perform tasks that are not performed by others around them – meaning that opportunities to enact diabetes management must be autonomously driven rather than reliant on the surrounding social environment and conventions of peers. This requires effort by CYPD/family and understanding from those around to reduce the awkwardness involved in needing to regularly undertake ‘unusual’ or counter cultural behaviours. Poor inter-personal, social/school, peer or family relationships or understanding of Type 1 diabetes can undermine CYPD efforts, impeding opportunities to enact diabetes management. Sensitivity, emotional support and understanding can better support the creation of opportunities to enactdiabetes control behaviours.  CYPD can find school environment difficult to navigate with regard to  diabetes control behaviours.  Families may lack social capital/assertiveness/communication skills to negotiate within local community for interventions to support CYPD and increase or make easier CYPD opportunities for diabetes management. | Understand what and how social/environmental factors impact individual self-care behaviours and blood glucose monitoring/ regulation.  Reduce inter-personal/family conflict and increase understanding of Type 1 diabetes needs, and incongruent beliefs about diabetes management to reduce emotional strain and increase ease and frequency of creating appropriate opportunities diabetes management. | **ENABLEMENT:**Provide skill training and practice to improve communication skills  **MODELLING:**Providing an example for CYPD to aspire to or imitate  **EDUCATION:**Increasing knowledge or understanding | **Individual level:**CYPD and family encouraged to explore diabetes management issues, through peer support/coaching structures. Promote advocacy/self-advocacy, and communication/mediation with school staff, peers, wider family and other parties who are part of social contexts where CYPD needs to feel able and comfortable to enact diabetes management behaviours.  Encourage CYPD to explore emotional responses to Type 1 diabetes management where focus is on supporting CYPD /families at the school/ community level to ease diabetes impacts on daily life. Enable and model inter-personal conflict resolution and assertiveness skills.  **Family / Community level:**Encourage families to find and use social networks and engage with wider family / community and school networks and agencies to enhance emotional and practical social support available to CYPD and family. Increase knowledge and understanding of Type 1 diabetes management in social and community networks. |
