# Supplementary material for: Codevelopment of a complex intervention to reduce inequalities in paediatric diabetes secondary care outcomes for children with type 1 diabetes from underserved groups
Source: BMJ Open. 2025 May 6;15(5):e089372. doi: 10.1136/bmjopen-2024-089372 (PMC12056613; doi:10.1136/bmjopen-2024-089372)
Supplement: online supplemental file 4 [file bmjopen-15-5-s004.docx]

**Supplementary files Table IV. APEASE criteria focus on Acceptability, Practicality and Equity following development of ‘Diversity in Diabetes’ intervention.**

| APEASE- Acceptability | YPAG x 1 / CYPD x 2 / family Focus x 2 groups | HCP Consultation - survey x2 | Stakeholder x 1 / Partners x 1 consultation meetings |
| --- | --- | --- | --- |
| Peer Support:  Life Coach  Family support worker  Clinic ethos / sessions | **Peer support:** was favoured by CYPD/ families face to face peer support structures to address issues identified in Phase 1.  **Life coach:** CYPD / families favoured a health and well-being coach role to support CYPD to prioritise, integrate and balance diabetes control behaviours in the broader context of their lives.  **Family support**: CYPD/families favoured the family support worker role to address /advocate for broader social and clinical issues when additional social input may be needed to reduce pressures on family life and support diabetes control.  **Clinic Ethos:** CYPD/families experienced communication which impacted negatively on their relationship with the diabetes team. CYPD / families also noted a lack of diversity among team members, CYPD /families strongly supported promotion of communication that offers CYPD and family members consultations that are respectful, positive, encouraging, and solution-focused.  **Additional clinic sessions**: CYPD and parents were supportive of the clinic offering more psychology support and making families aware of its availability of this support. CYPD and families were also reported that their time with the diabetes team is already pressured. | **Peer support:** favoured by HCPs to address alienation of CYPD  **Life coach**: favoured by HCPs but should be managed within the diabetes MDT to ensure coaches understand the overarching aims of the service and to maintain good communication with the clinical team.    **Family support:** HCPs considered it important for a family/community worker to be integrated into the team. Will bring useful insights that support a holistic approach to care.    **Clinic ethos**: was in line with current service provision, and most respondents were already engaging in training focused on the ethos of the clinic and communication with CYPD/families from diverse backgrounds    Need to ensure 1-1 support and effective CYPD/family education in diabetes control  **Additional clinic sessions****:** Many respondents felt that their service was already running appropriate sessions, or that they needed more resources (staffing and time) to be able to deliver additional sessions and groups. | **Peer support:** was favoured and could be integrated with Diabetes UK / Together with Diabetes ‘Young Leaders’ programme and consistent with NHS England ‘6 principles of good peer support’ based on shared experiences of people living with Type 1 diabetes  **Life coach:** was favoured as dedicated diabetes health and well-being coaches are in place in some localities, including some with a focus on reaching diverse and underserved groups.  **Family support:** Stakeholders / HCPs noted that role is already performed in many diabetes teams, so there are existing models for employment within MDT.  **Clinic ethos:** clinical excellence/quality improvement work ongoing within all clinics. Feedback to teams on these communication issues, related specifically to underserved/diverse groups, may be helpful. Negative encounters with clinicians (despite being recognised as potentially inappropriate/unhelpful) are understood in the context of genuine clinical fear for CYPD health/life/safety - that is the communication of critical concerns is clinically warranted, even if the communication style may be unhelpful. |
| APEASE-Practicality | **YPAG x 1 / CYPD x2 / family Focus x2 groups** | **HCP Consultation - survey x 2** | **Stakeholder x 1 / Partners x 1 consultation meetings** |
| Peer Support:  Life Coach  Family support worker  Clinic ethos / sessions | **Peer support:** CYPD were interested to shape programmes focusing on specific peer-led relevant diabetes control issues (e.g. adaptation to new technology increased, physical activity, family dynamics, school and socio-emotional issues).  **Life coach:** CYPD focused on life skills and developing self-advocacy and the chance to discuss life issues and the underlying barriers to good self- care beyond the technicalities of diabetes control. Engagement more likely outside of hospital setting.  **Family support:** CYPD’ parents were however wary about the burden of increased contacts with social/health teams where contact with the diabetes team was already extensive/demanding on family life.  **Clinic ethos:** CYPD and CYPD’ parents noted a lack of diversity among team members, and communication experienced as patronising/judgemental and unsympathetic (of life circumstances). CYPD and parents strongly supported promotion of communication that offers CYPD and family members consultations that are respectful, positive, encouraging, and solution focused.  **Additional clinic sessions**: CYPD/ Families do not necessarily wish to, nor are practically able to spend more time at the clinic (e.g. given work, other siblings/dependents etc). | **Peer support** currently facilitated within MDT / by family support workers. It could work in partnership with other providers in that area such as youth services, arts and sports organisations. Parents would benefit from accessing facilitated peer support.  .  **Life coach:** It was noted that life coaching for younger CYPD would work best at a parent or family level, rather than at an individual CYPD level.- Some respondents felt that CYPD more likely to engage if they were seen as independent of the team and if they met the CYPD outside of the hospital setting.  **Family support:** Many teams already employ family/community workers. However, some parents’ prefer not to have another diabetes/social practitioner involved regularly in already pressured everyday life.  Some clinics had youth workers in place for those who are at risk of disengaging or complex issues that the clinical team lack the resources to support.  **Additional clinic sessions:** MDTs feel pressured with limited time to accomplish roles; asking more of the teams would need to be resourced, though much of this work is supported and already performed in many teams. | **Peer support:** Youth Workers are already facilitating peer support in some places (e.g. setting up local peer group social sessions and working on mentoring and empowerment schemes (YES), working with CYPD to support engagement in peer support) but coverage is not consistent across UK. Peer support would be complementary, working hand in hand with health and social care providers and will have impact beyond the peer relationships and will enhance communication with other intervention roles.  **Life coach**: Training needed to enable diabetes specialist youth or family workers or psychologists to provide this intervention alongside other work. ‘Youth Empowerment and Skills (YES)’ programme, ‘Tree of Life’ programme offer relevant co-created and evaluated approaches to supporting CYPD in these areas, in ways that also increase CYPD peer contact and support.  **Family support:** There can be difficulties with engagement and uptake for families with multiple additional demands (e.g. large families, other health conditions, negative associations with social services).  **Clinic ethos:** ongoing clinical excellence/quality improvement work within all clinics. Feedback to teams on these communication issues, related specifically to underserved/diverse groups, may be helpful.  **Additional clinic sessions:** HCPs already engage in person-centred training, but supported the need for continuous improvement and welcomed specific input related to sensitivity and communication in work with CYPD from diverse and economically deprived backgrounds to enhance treatment concordance. |
| APEASE- Equity | **YPAG x 1 / CYPD x2 / family Focus x2 groups** | **HCP Consultation -survey x 2** | **Stakeholder / Partners consultation meetings** |
| Peer Support:  Life Coach  Family support worker  Clinic ethos / sessions | **Additional sessions:** It was noted that offering additional clinical support would not necessarily lead to change. However, a focus on motivation/barriers/values etc in the context of social issues could be beneficial (more in line with life coaching or family or youth worker roles). | **Peer support:**  Concerns for peer support related to prior experiences of low engagement, particularly from underserved groups. Keeping it local would enable the service to better understand and meet the needs of young people in that area so as to limit exclusion due to issues such as transport.  **Clinic ethos:** HCPs described commonly addressing financial issues related to disability benefits/technology use, and an openness with patients and amongst the MDT team in raising  **Additional clinic sessions:** There was a shared concern around the demands placed on families and reaching diverse underserved groups who find it hardest to attend clinic or spend any longer than they already do with the MDT in clinical settings, given other responsibilities and pressures. | **Family support:** CYPD / families from underserved groups are not always those reached therefore targeting of additional support in this area would need to be carefully monitored.  **Additional clinic sessions:** Conflict in additional sessions  as concerns related to clinical pressures on time and resources and the need for additional funded time to allow for more sensitive (and potentially time-consuming) work.  Disadvantaged families already struggle to attend allocated appointments so may be least likely to benefit from additional sessions |
