# Supplementary material for: Codevelopment of a complex intervention to reduce inequalities in paediatric diabetes secondary care outcomes for children with type 1 diabetes from underserved groups
Source: BMJ Open. 2025 May 6;15(5):e089372. doi: 10.1136/bmjopen-2024-089372 (PMC12056613; doi:10.1136/bmjopen-2024-089372)
Supplement: Supplementary file 5 [file bmjopen-2024-089372.reviewer_comments.pdf]

PEER REVIEW HISTORY

BMJ Open publishes all reviews undertaken for accepted manuscripts. Reviewers are asked to complete a checklist review form and are provided with free text boxes to elaborate on their assessment. These free text comments are reproduced below.

ARTICLE DETAILS

Title (Provisional)

Co-development of a complex intervention to reduce inequalities in paediatric diabetes secondary care outcomes for children with type 1 diabetes from underserved groups

Authors

Searle, Aidan; Wheeler, Jessica; Litchfield, Ian; Shield, Julian PH; Barrett, Timothy; Greenfield, Sheila; Leeson-Beevers, Kerry; Redwood, Sabi; Consortium Members, The Diversity in Diabetes

---

VERSION 1 - REVIEW

**Reviewer**	**1**
**Name**	**Tremblay, Elise**
**Affiliation**	**Boston Children's Hospital**
**Date**	**04-Sep-2024**
**COI**	**None**

Introduction: Sets the stage for the paper nicely, establishes why this question is important. Minor comments below.

-Define MRC

-May be helpful to delineate more clearly that you are giving a summary of earlier phases that are published and complete before going into the details of Phase 1 (it sounds like you are getting into a methods section). This summary also seems somewhat longer than would be expected for an introduction to a paper.

Research Design and Methods: Overall it is a little challenging to get a good picture of the overall methods. May be useful to include a summary up front of the methods (i.e. we relied upon qualitative interviews - how did you analyze data? - and focus groups to answer key study questions). It is unclear from your description how you collated the data (i.e. thematic analysis, did you achieve saturation in your interviews?) and how you selected the participants.

Minor issues:

-Define APEASE criteria earlier

Results:

-May be helpful to include some quotes from qualitative interviews to demonstrate some of your summary points.

-Element 1 description of the elements of intervention you defined is great; I like understanding where the ideas came from and linking them back to the qualitative data. Element 2-3 does not contain this same explanation and you may also want a reference for the role of life coaching. Element 4 again has a good description of source of the idea for the intervention, but does not define what the intervention would be as well. Table 1 has a LOT of information and may be better to split up.

Discussion: First summary paragraph is very clear, but then the discussion loses some focus. Inclusion of the role of under resourced families is very important; discussion around this is good. Should have more formal inclusion of strengths and limitations. Conclusion is weak.

Overall: incredibly important area to study and the manuscript demonstrates a lot of work; improving the clarity with which the methods and results are described will strengthen the manuscript and make it more accessible.

**Reviewer**	**2**
**Name**	**Everett, Estelle**
**Affiliation**	**University of California Los Angeles**
**Date**	**03-Oct-2024**
**COI**	**none**

This paper nicely describes a multi-level intervention to address disparities in the pediatric T1D population.

Strengths:

- the foundation of these intervention are well rooted in established frameworks which guided the therapy and development of the intervention

- interventions informed by feedback from patient, families and providers

- intervention was multi-leveled and aimed to target both clinical and social determinants of health

Limitations:

- there was little description of the demographic breakdown (ethnicity, primary language, socioeconomic status etc) of the study population, so it is unclear what population these interventions were co-developed with and are most relevant too.

- similarly limited description of HCP (e.g. which type of providers etc).

- While type of intervention are described, and the acceptability of these interventions. I would have like some details what these interventions would look like in practice.

VERSION 1 - AUTHOR RESPONSE

Responses to reviewers:

Reviewer: 1
Dr. Elise Tremblay, Boston Children's Hospital
Comments to the Author:
Introduction: Sets the stage for the paper nicely, establishes why this question is important. Minor comments below.
-Define MRC
Now defined on first mention [Medical Research Council]

-May be helpful to delineate more clearly that you are giving a summary of earlier phases that are published and complete before going into the details of Phase 1 (it sounds like you are getting into a methods section). This summary also seems somewhat longer than would be expected for an introduction to a paper.

Introduction is now thoroughly reworked for clarity and conciseness, including in relation to the summary of relevant background literature that situates the Diversity in Diabetes Study, and also for phases 1 and 2 (that is, phase 1 includes information on the Burden of Treatment Theory, which foregrounds the contextual focus of this intervention co-development work; phase 2 introduces the COM-B). It also briefly outlines and makes clear the content of the report.

Research Design and Methods: Overall it is a little challenging to get a good picture of the overall methods. May be useful to include a summary up front of the methods (i.e. we relied upon qualitative interviews - how did you analyze data? - and focus groups to answer key study questions). It is unclear from your description how you collated the data (i.e. thematic analysis, did you achieve saturation in your interviews?) and how you selected the participants.

Methods thoroughly reworked for clarity and to ensure sufficient details for reproducibility of methods, with attention to Phase 1 selection of participants, empirical methods and analysis, and additional detail for Phases 2 and 3 focus groups and COM-B/APPEASE integration process (including HCP survey methods). The overall methods are summarised at the outset, with a figure of the overall process provided, and the methods sections split into distinct phases.

Minor issues:
-Define APEASE criteria earlier

Defined on first mention

Results:
-May be helpful to include some quotes from qualitative interviews to demonstrate some of your summary points.

Additional detail is now provided to explain the link between qualitative interview findings (relevant analytical themes) and intervention elements. This paper is not focused on the empirical work, as it is an overview of phases and COM-B intervention development, so we have not included verbatim quotes, but we have summarised relevant analytic themes from Phase 1 empirical work, with descriptions.

-Element 1 description of the elements of intervention you defined is great; I like understanding where the ideas came from and linking them back to the qualitative data. Element 2-3 does not contain this same explanation and you may also want a reference for the role of life coaching. Element 4 again has a good description of source of the idea for the intervention, but does not define what the intervention would be as well.

Elements 2, 3 and 4 have been developed to increase the clarity and level of explanation, so these are in line with the detail provided for element 1, and linked back to Phase 1 qualitative thematic analysis throughout. References for health and well-being coaching are now included.

Table 1 has a LOT of information and may be better to split up.

Table 1 is now split into three tables– given additional detail and explanation now provided elsewhere in the results (e.g. descriptions of elements 1-4 as above), we now offer these three tables as supplementary files (supplementary files Tables I-III) which offer additional detail and insight to the mapping process. We felt the paper is complete and more readable, without directly including these.

Discussion: First summary paragraph is very clear, but then the discussion loses some focus. Inclusion of the role of under resourced families is very important; discussion around this is good. Should have more formal inclusion of strengths and limitations.

Discussion is thoroughly reworked for clarity. The first paragraph summary is retained, though improved for completeness and conciseness. The discussion is now structured as follows:

- second paragraph - integration with relevant wider literature around peer support, family support, health and well-being coaching, and enhanced communication and shared understanding - touching on the relevance of shared backgrounds, as highlighted by participants;
- third paragraph - the need to move from narrow focus on biomedical discourses to broader engagement and understanding relevant to diabetes management in diverse contexts;

- fourth paragraph - the role and need for effective communication and the ways this can be prone to erosion in clinical work with underserved communities;
- fifth paragraph - the significance of material and social circumstances and life events over 'cultural factors' and the role of the PPIE in clarifying this;
- sixth paragraph - briefly, strengths and limitations;
- seventh paragraph - implications for paediatric diabetes health services – recognising that the interventions co-developed through this work were welcomed and exist in some teams, but that coverage is uneven and this work justifies funding that recognises the relevance and importance of broadening the remit of paediatric diabetes multi-disciplinary teams;
- eight paragraph/conclusion - we cannot hope to solve structural inequalities through secondary care service provisions, but we can hope to strengthen patients in relation to the impacts of the structural circumstances to which they are exposed, and provide a level of input that serves to help build welcome social networks (through peer support) and through advocacy and empowerment work to increase patients confidence and ability to engage effectively with health services and local community providers in ways to better ensure health related adaptations as well as better understanding and engagement with available treatments and services.

We recognise this is slightly over the five paragraphs, but also that this preference is not a requirement – however, we hope the 8 paragraphs are sufficiently concise and that each justifies inclusion.

Conclusion is weak.

See above, discussion has been reworked and stronger conclusion brought in – we hope the entire discussion and conclusion is significantly strengthened. We have added further references as part of the reworking of the paper and integration within the wider literature.

Overall: incredibly important area to study and the manuscript demonstrates a lot of work; improving the clarity with which the methods and results are described will strengthen the manuscript and make it more accessible.

We believe this report is now strengthened with increased clarity throughout.

Reviewer: 2
Dr. Estelle Everett, University of California Los Angeles
Comments to the Author:
This paper nicely describes a multi-level intervention to address disparities in the pediatric T1D population.

Strengths:
- the foundation of these intervention are well rooted in established frameworks which guided the therapy and development of the intervention

- interventions informed by feedback from patient, families and providers
- intervention was multi-leveled and aimed to target both clinical and social determinants of health

Limitations:
- there was little description of the demographic breakdown (ethnicity, primary language, socioeconomic status etc) of the study population, so it is unclear what population these interventions were co-developed with and are most relevant too.

- similarly limited description of HCP (e.g. which type of providers etc).

Results Table 1 now provides clear description of the demographic breakdown of the Phase 1 study population. It is hopefully now entirely transparent which populations these interventions were co-developed with and are potentially most relevant too. In addition, a breakdown of the practitioner roles of HCP survey 1 respondents is now given.

- While type of intervention are described, and the acceptability of these interventions. I would have like some details what these interventions would look like in practice.

Interventions are more clearly and thoroughly outlined in the results sections (as above), in particular in descriptions of elements 1-4 so that what they comprise of is made clear. Additional references that relate to health and wellbeing coaching are also provided to clarify the nature of this intervention for readers who may not be familiar with it (as above).

Reviewer: 1
If you have selected 'Yes' above, please provide details of any competing interests.: None

Reviewer: 2
If you have selected 'Yes' above, please provide details of any competing interests.: none

We hope this fully addresses all the identified issues and that following the reviewers positive comments, and our amendments to all of the issues they have identified, this report is now ready for publication with BMJ Open.

In addition, we would like to point out that we have added in an additional co-author (acknowledged in the paper as originally submitted, Kerry Leeson-Beevers is now included as co-author – KLB is the Diversity in Diabetes PPIE Lead and has been an integral part of the project and co-development work, contributing throughout to all phases, but had been unable to review the first draft of this paper before its submission - KLB is now appropriately included as co-author having reviewed and commented on the current draft)

Many thanks for your work in progressing this publication.
